# Economic growth, human capital, and energy consumption in Algeria: evidence from cointegrating polynomial regression and a simultaneous equations model

**DOI:** 10.1007/s11356-022-23657-7

**Published:** 2022-11-02

**Authors:** Mohammed Bouznit, María P. Pablo-Romero, Antonio Sánchez-Braza

**Affiliations:** 1 Laboratoire Economie et Développement, Faculté des Sciences Economiques, Commerciales et des Sciences de Gestion, Université de Bejaia, 06000 Bejaia, Algeria; 2 Department of Economic Analysis and Political Economy, Faculty of Economics and Business Sciences, Universidad de Sevilla, Avda. Ramón y Cajal 1, 41018 Seville, Spain

**Keywords:** Human capital, Energy consumption, Cointegrating polynomial regressions, Simultaneous equations model, Energy-EKC hypothesis, Algeria

## Abstract

This article aims to empirically analyze the direct and the indirect effects of human capital on energy consumption in Algeria, as well as to test the possible presence of the energy-environmental Kuznets curve (E-EKC) hypothesis, over the period 1970–2017, using cointegrating polynomial regressions (CPR) with break points, and a simultaneous equations model. The obtained results indicate that human capital directly reduces energy demand, and indirectly increases it through income and physical capital stock channels. However, the direct effect is higher than that of the indirect effect. Additionally, CPR results confirm a monotonic increasing relationship between energy use and real GDP per capita; therefore, there is no evidence of the E-EKC hypothesis. This means that increasing economic growth leads to a rise in energy consumption and, in turn, to an intensification of CO_2_ emissions. The results also indicate that physical capital stock per capita, urban population ratio, and real GDP per capita are positively linked to energy use per capita. In that context, it may be appropriate to adapt the energy system to the growing demand, promoting greater use of renewable energies, if emissions growth is to be contained. Investment in education and improving the quality of human capital is a good way for Algeria to reduce energy consumption and protect the environment, without negatively impacting economic growth.

## Introduction

The growth in greenhouse gas (GHG) emissions is mainly caused by energy consumption and its increase over time (Omri 2013; Saboori and Sulaiman 2013; Kasman and Duman 2015). According to Sorrell (2015), the worldwide energy demand has continually increased, with an annual growth rate of 2.5%, since 1850. Hence, improving energy efficiency and reducing dirty energy demand are the most appropriate means to mitigate climate change. Nevertheless, this can only be achieved once the determinants of energy consumption are clearly identified. An extensive research field has been developed along this line, highlighting the relationships between different variables, as pointed out in the literature review section.

On the one hand, many authors have highlighted that income is a main factor which explains energy demand. Therefore, economic factors affecting income could indirectly determine energy demand, for instance, physical and human capital, generating an income effect (Salim et al. 2017; Yao et al. 2019). On the other hand, other authors have highlighted the role of energy as a productive factor, analyzing the substitutability or complementarity, with respect to other factors, such as human and physical capital. Therefore, these other factors could affect the energy demand in the productive process. Finally, some authors have combined both perspectives, allowing the direct and indirect effects of human and physical capital on energy consumption to be studied, as in Yao et al. (2019). Nevertheless, the results are still inconclusive and may vary between countries.

Following these last studies, the aim of this work is to empirically analyze the direct and indirect effects of human capital on energy consumption in Algeria, which is a novelty in the literature. The study of this country is especially interesting as Algeria is one of the most energy-consuming, and highest CO_2_ emitters, among developing countries (Bouznit et al. 2020). Likewise, according to the data from the World Bank (2021) database, its annual growth rate over the period 1970 to 2017 was 10.67%, while the rate worldwide was only 0.99%. Therefore, in order to be able to promote economic measures for reducing energy consumption, and to achieve the targets set in Algeria’s Intended Nationally Determined Contribution (Algeria’s INDC-UNFCCC 2015), it is necessary to know how the relationships, between energy consumption and economic growth, have occurred. This fact also seems especially important in a country that is very vulnerable to multiple forms of climate change. In this sense, an analysis that enables an assessment of the direct and indirect effects may provide important information. This would assist policymakers to implement a suitable governmental strategy, adequate measures to rationalize energy consumption, and promote energy saving practices.

With this objective, the methodology by Yao et al. (2019) is applied. However, some differences are introduced in the model. Firstly, the direct effect is analyzed through a non-linear energy demand function, contrasting the energy-environmental Kuznets curve (E-EKC) hypothesis, extended with human capital and urban density, using the cointegrating polynomial regressions (CPR) method. This method offers more suitable solutions than the linear cointegration method to model the non-linear relationship between the studied variables, as it addresses endogeneity and errors of serial correlation. To our knowledge, no study has been made which adopts the CPR method to test the EKC, for the case of developing countries, nor to test the E-EKC. Therefore, this research may provide interesting results for the literature, as previous studies have shown that, when studied using this method, only a reduced number of the developed countries have been supported by the EKC hypothesis (Wagner 2015; Wagner and Hong 2016).

Secondly, the indirect effects are determined through a system of equations linked to the production process, as in Yao et al. (2019), where the production function is defined as a Cobb–Douglas function, extended to human capital and oil prices, the last variable being included to take into account the special circumstance of Algerian production. Among the other functions included in the system of equations, is one in which human capital depends on physical capital, allowing the analysis of the relationships of complementarity or substitutability between human and physical capital. For this, time series econometric techniques are used, referring to the period of 1970–2017.

Therefore, the main novelties of this paper are as follows: analysis of the direct and indirect effects of human capital on energy consumption in Algeria; estimation of the direct effects of human capital on energy consumption using a nonlinear energy demand function instead of the linear function, previously used in Yao et al. (2019) and Salim et al. (2017); adoption of the CPR method to estimate this nonlinear function, which has not been previously used to test the E-EKC hypothesis; and the inclusion of a function in which human capital depends on physical capital in the system of equations used to study the indirect effects of human capital, in order to fully study the substitutability or complementarity effects between productive factors.

The remainder of this paper is structured as follows. “Literature review” section presents a literature review. The “Methodology” section details the methodology. The data used and descriptive analysis are presented in “Data and descriptive analysis” section. The “Results and discussion” section gives the results and discussion and, finally, the main conclusions are offered in “Conclusions and policy implications” section.

## Literature review

Factors influencing energy consumption have been extensively studied from different perspectives. However, although an extensive research field has been developed along this line, the relationships between some factors affecting energy consumption are still inconclusive.

From the seminal study by Kraft and Kraft (1978), one branch of the studies analyzing energy consumption has focused on analyzing its relationship with economic growth. Although the results of these studies do not always find the same causal relationship for the variables (Coers and Sanders 2013; Pablo-Romero and Sánchez-Braza 2015), many of them show that economic growth causes increased energy consumption, as shown in the recent review of these studies by Tiba and Omri (2017). Likewise, focusing on MENA countries, the review by Gorus and Aydin (2019) also finds mixed results in the studies, but most confirm the causal relationship of economic growth to energy consumption. Thus, many authors have highlighted that income is a main factor which explains energy demand.

However, some authors emphasize that income does not always affect the energy demand with the same intensity, considering that the relationship between the variables may be non-linear, with even the sign of the relationship varying among other causes, due to the income level. Thus, an extension of the energy demand studies has been developed, by comparing the so-called E-EKC hypothesis. Among others, studies by Pablo-Romero and De Jesús (2016) and Dong and Hao (2018) may be cited. This non-linear income effect on energy demand means that both income level and economic growth may affect energy consumption. Hence, factors that determine the income level and induce economic growth, indirectly, are affecting energy consumption. Thus, the factors of physical and human capital, which positively affect income level and economic growth, according to the endogenous growth theory (see for example Romer 1990 and Barro 2001), would be affecting energy demand, in what has been called the income effect (Salim et al. 2017; Yao et al. 2019).

Another branch of the studies, related to energy consumption, has focused on the study of the relationships between production and energy demand by focusing on the production functions, considering the last variable as an additional productive factor. Some studies consider energy as an additional productive factor, without analyzing the relationship with the other productive factors. Additionally, some studies, such as those by Moroney (1992), Sharma (2010), Dieck-Assad and Peralta (2013), Fang and Chang (2016), and Fang and Chen (2017), find that energy has a positive impact on production. Other studies also consider the relationships between energy and other productive factors. Following the study by Berndt and Wood (1979), some have focused on the study of the substitutability between energy and capital, considering that the implementation of energy-saving equipment may correspond to a substitution of capital for energy (Kim and Heo 2013). However, the empirical literature does not show consensus on the substitutability hypothesis between both factors (Lin and Xie 2014). More recently, other studies have focused on the substitutability or complementarity between energy and human capital, so that if substitutability is found, the increase in human capital reduces energy demand (Pablo-Romero and Sánchez-Braza 2015; Fang and Yu 2020). The results obtained so far are not conclusive and more research is needed (Salim et al. 2017). Taking into account these relationships between productive factors (including energy), some authors, such as Salim et al. (2017) and Yao et al. (2019), have considered that physical and human capital can have effects on energy consumption by means of physical capital investment effects.

Focusing on the effects of human capital on energy consumption, several studies have analyzed their relationships, from an economic growth or energy demand perspective (a summary of these studies is presented in Table 1). Some focused on the causality between human capital, energy, growth, and other variables (Chen and Fang 2018; Azam 2019; Shahbaz et al. 2019; Bashir et al. 2019; Fang and Wolski 2021), others analyzed the causality differentiating between renewable and non-renewable energy (Hanif et al. 2020), or only studying these effects on renewable energies (Sarkodie et al. 2020; Wang et al. 2020a; Ozcan and Danish 2022). Additionally, some of them have studied the interaction between the energy consumption and the human capital in production functions (Pablo-Romero and Sánchez-Braza 2015; Fang and Yu 2020), while other studies have included the human capital variable as a relevant factor in the energy demand function (Salim et al., 2017; Akram et al., 2020). Among the latter, some of them have focused on the effect of human capital on nonrenewable energy consumption (Alvarado et al. 2021), on clean and dirty energies (Shahbaz et al. 2022), and on the sectoral electricity energy demand (Pablo-Romero et al. 2022). Others have focused on the human capital asymmetric effects (Sehrawat 2021) and on the scale, technical, and structural human effects (Wang et al. 2022).

**Table 1:** Summary of empirical studies on the effect of human capital on energy consumption

Author(s)	Countries/regions	Sample period	Variables	Methodology	Main findings
Pablo-Romero and Sánchez-Braza (2015)	38 major countries: OECD, BRICS, East Asia, NAFTA, East European, EU	1995–2007	Gross value added, human capital index, physical capital stock, productive energy use, total employee hours	Panel data GMM (Translog function)	Human capital, physical capital, and energy positively affect gross value added. Weak substitutability relationship between physical capital and energy. Important substitutability between energy and human capital
Salim et al. (2017)	China: 19 Provinces	1990–2010	Energy consumption (EC), GDP, energy price, capital stock, human capital (HC)	DOLS, FMOLS, AMG, PMG, DFE structural equation system	Negative relationship between HC and EC in China. Heterogeneous relationships across provinces
Chen and Fang (2018)	210 Chinese cities and several sub-samples	2003–2012	Total output, investment in physical capital, investment in human capital, industrial electricity consumption	Continuously-updated fully modified panel estimation Granger causality test	Electricity positively affects output. Bi-directional causal relationship between human capital investment and electricity consumption in the whole sample. Different results in sub-samples
Yao et al. (2019)	OECD	1965–2014	Total energy consumption per capita, fossil energy and clean energy consumption per capita, human capital, physical capital, per capita real income, proxy of energy prices, ratio of investment to capital stock, ratio of net energy imports to total energy consumption	Panel cointegration, panel regression using OLS and AMG, simultaneous equations model	Human capital impacts negatively on total and fossil energy consumption, and positively on clean energy consumption
Azam (2019)	BRICS	1981–2015	GDP, energy use, environmental pollution, physical capital, human capital, financial development	FMOLS, Robust Least Squares, D-H causality test	Energy consumption can influence human capital
Shahbaz et al. (2019)	USA	1975–2016	Energy use per capita, consumption, education, real GDP per capita, natural resources, crude oil prices, exports diversification	Bootstrapping ARDL approach, VECM’s Granger causality (Time series)	Education negatively causes energy use, feedback effect between education and energy
Bashir et al. (2019)	Indonesia	1985–2017	GDP, CO_2_, energy consumption, human capital	VECM estimates	Energy consumption has insignificant effect on human capital and human capital on energy consumption in short and long run
Hanif et al. (2020)	16 OECD and 14 non-OECD emerging countries	1990–2017	Renewable energy (RE) and Non-renewable energy consumption (NRE), Technology innovation, Human Development (HD), Exchange rate, Oil prices	Feasible Generalized Least Square	Inverted U-shaped relationship between NRE and HD. U-shaped relationship between RE and HD
Wang et al. (2020a)	BRICS	1990–2015	Human development index, GDP, biomass energy consumption, industrialization, FDI, trade openness	Panel data	Biomass energy consumption (BEC) increases human development (HD) Bidirectional causality between HD and BEC
Sarkodie et al. (2020)	China	1961–2016	CO_2_ emissions, Human capital index, Renewable and fossil energy consumption, trade, GDP per capita	Dynamic ARDL simulation (Time series	Human capital and fossil energy consumption are catalysts of climate change
Akram et al. (2020)	73 countries: Asia, Africa, Europe, Latin America	1990–2014	Energy consumption per capita, real GDP per capita, Energy price, Real per capita capital stock, human capital	Dynamic panel data (GMM)	Human capital negatively affects energy consumption
Fang and Yu (2020)	56 countries and several sub-samples	1970–2014	GDP, physical capital, human capital, energy	Continuously updated fully modified method	Complementarity relationship between energy and human capital
Fang and Wolski (2021)	China	1965–2014	GDP, physical capital, Human capital (HC), total energy consumption (EC) and coal, oil natural gas and hydroelectricity	Linear and non-linear causal relationships	No causal links between energy and economic growth. In linear model, substitution effect between HC and EC and/coal. No link between the variables in the non-linear model
Alvarado et al. (2021)	OECD countries	1980–2015	Non-renewable energy consumption, real per capita output, and human capital index, globalization index, urban population, added value of services	Threshold regressions, second generation cointegration techniques, FMOLS, and causality	The accumulation of human capital leads to reduced non-renewable energy consumption
Sehrawat (2021)	India	1970–2014	Energy consumption, human capital, income inequality, GDP, physical capital, energy price	Non-linear ARDL	Human capital lowers the energy demand
Shahbaz et al. (2022)	China	1971–2018	Overall energy demand, dirty energy demand, clean energy demand, GDP, human capital, imported energy, R&D expenditures	ARDL model with structural breaks	Human capital and R&D are negatively linked to three types of energy consumption, GDP positively affects the three types of energy consumption
Wang et al. (2022)	30 provincial regions of China	1997–2018	Energy consumption per capita, real average human capital, GDP per capita, technological progress in the energy field, Theil index, urbanization, export, and energy price	STIRPAT model	The scale effect of human capital increases energy consumption, the technical and structural effects of human capital negatively affect energy consumption
Ozcan and Danish (2022)	Turkey	1980–2017	Renewable energy, globalization, GDP, human capital	Dynamic ARDL simulation	Human capital stock causes renewable energy consumption
Pablo-Romero et al. (2022)	17 Autonomous Communities of Spain	2000–2013	Electricity consumption by productive sectors, Gross Value Added, human capital, productive physical capital, temperature indexes	Panel data: Feasible Generalized Least Squares	Human capital has negative effects on electricity consumption, except for the public administration sector

Prepared by the authors on the basis of the extant literature

Finally, a mixed study, considering both the energy demand function and production function perspective, has been conducted by Salim et al. (2017) and Yao et al. (2019). On the one hand, the study by Salim et al. (2017) considers the possible income effect and its relationship with physical capital, using a structural equation system composed of three models, where energy consumption, income, and capital stock are the dependent variables. On the other hand, in order to take into account and assess the different effects of human capital on energy demand, in Yao et al. (2019), a simultaneous equation model is employed. In that study, the authors analyze two effects of human capital on energy demand, the direct and the indirect effect. While the direct effect of human capital on energy demand is studied by means of an energy demand function, the indirect effect (related to the income and other effects) is studied by means of structural equations that reflect the production process.

The results of these studies are not conclusive as, in some cases, human capital negatively affects energy consumption (for example in Shahbaz et al. 2019), in other cases, it has no affect (for example in some estimates in Fang and Wolski 2021) and, in others, it affects positively (for example in Fang and Yu 2020). Some authors considered that this different effect could be explained by different development levels (Arbex and Perobelli 2010; Salim et al. 2017). Additionally, it could also be explained by the predominance of the direct or indirect effect indicated by Yao et al. (2019), since both effects are of opposite signs. Thus, it may be interesting to assess these effects in different economies.

## Methodology

This study analyzes the effect of human capital on total energy use in Algeria, over the period 1970–2017, for which sufficient data are available. Following the study by Yao et al. (2019), two effects are studied: the direct and the indirect effect. The direct effect of human capital on energy demand is studied by means of an energy demand function, and the indirect effect (related to the income and other effects) is studied by means of structural equations that reflect the production process.

### Direct effect of human capital on total energy use and E-EKC hypothesis

#### Model

As stated before, the direct effects of human capital on energy use is studied through the empirical estimation of an energy demand function, where human capital is an additional explanatory variable, in line with recent papers, such as those by Salim et al. (2017), Yao et al. (2019), and Akram et al. (2020).

The extended energy demand function used in this study may be expressed as follows:

1 $$EU=F\;\left(Y,H,UP\right)$$

where *EU* is energy use measured as kg of oil equivalent per capita expressed in logarithms, *Y* is real GDP per capita at constant 2011 national prices (in millions of 2011 US$) expressed in logarithms, *H* is the human capital index expressed in logarithms, based on average years of schooling and returns to education, and *UP* is urban population (% of total population) expressed in logarithms. The data used are annual figures covering the period 1970–2017 and taken from three databases, namely World Development Indicators (World Bank 2021), Penn World Table 9.1 (Feenstra et al. 2015, 2021), and BP Statistical Review of World Energy (2020).

**Fig. 1:**
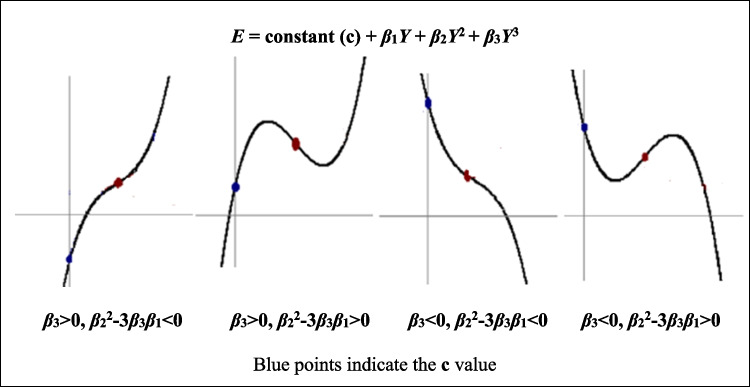
Representation of the energy demand cubed function depending on the value of the parameter

Three considerations related to this energy demand function could be explained. Firstly, recent studies have been pointing out that the influence of income on energy demand is not linear. Therefore, the squared and cubed values of the income variable have been added to explanatory variables (for example in Yin et al. 2015; Bouznit et al. 2018; Pablo-Romero et al. 2019). The incorporation of these variables allows testing of the E-EKC hypothesis, and the identification of the energy consumption tendency, when income per capita grows by means of the signs of the parameters related to income and the squared and cubed variables. Details of possible cases, related to these parameters’ signs, can be found in Dinda (2004). However, it is worth highlighting how the relationships, between energy demand and income, may be depending on the cubed parameter value and its relation to the other parameter values. Figure 1 shows these possible cases, where the energy demand function (*E*) depends on income (*Y*), squared income (*Y*^2^), and cubed income (*Y*^3^).

Secondly, the urban population has also been widely considered in energy demand studies. A detailed review of these studies can be found in Shahbaz et al. (2017). Some of these studies focus on total energy demand (for example, Cole and Neumayer 2004), while others focus on its effect on a type of energy. For example, Dhal & Erdogan (1994) studied its effect on oil consumption, while Mickieka & Fletcher (2012) tested its effect on coal. Other studies focus on specific sectors. Some authors, such as Wang (2014), analyze the relationship between urbanization and energy demand in the residential sector, or in the transport sector, such as in Lee et al. (2017). The results obtained in these studies are diverse. Some studies show a positive effect of urbanization on energy consumption (for example in Lui 2009), others observe a negative effect (for example in Liu et al. 2017), while others, such as Cole & Neumayer (2004) and Duan et al. (2008), find a U-shaped relationship between both variables. Likewise, evidence has been found that the effect is not similar in different countries or regions (for example in Zhang & Lin 2012).

Thirdly, energy prices have not been included in the energy demand function, on the basis that energy prices are very low and greatly subsidized by the Algerian authorities (Bouznit et al. 2018). Thus, the increasing prices in a period may be captured by the time variable, included in the model.

Therefore, the functional form of energy demand can be expressed as follows:

2 $${EU}_{t}=c+{\sum }_{k}{\theta }_{k}{D}_{t,k}+{\delta }_{t}+{\beta }_{1}{Y}_{t}+{\beta }_{2}{Y}_{t}^{2}+{\beta }_{3}{Y}_{t}^{3}+{\beta }_{4}{H}_{t}+{\beta }_{5}{UP}_{t}+{\varepsilon }_{t}$$

where *t* is a linear time trend, *c* is an intercept, and *ε*_*t*_ is a white-noise error term. *D*_*t,k*_ is a dummy variable that captures the regime changes in the model. Finally, *c*, *θ*_*k*_, *δ*, *β*_1_, *β*_2_, *β*_3_, *β*_4_, and *β*_5_ are the parameters to be estimated.

It is worth noting that including *Y*, *Y*^2^, and *Y*^3^ in Eq. (2) may generate multicollinearity among them. To avoid this problem, Eq. (2) is rewritten by transforming the studied times series into their mean centered values, as in previous literature (Pablo-Romero & Sánchez-Braza 2017; Bouznit et al. 2018). Lowercase variables are used to indicate that change. Hence, Eq. (2) may be rewritten as follows:

3 $${eu}_{t}=c+{\sum }_{k}{\theta }_{k}{D}_{t,k}+{\delta }_{t}+{\beta }_{1}{y}_{t}+{\beta }_{2}{y}_{t}^{2}+{\beta }_{3}{y}_{t}^{3}+{\beta }_{4}{h}_{t}+{\beta }_{5}{up}_{t}+{\varepsilon }_{t}$$

The signs and statistical significance of the estimated coefficients *β*_1_, *β*_2_, and *β*_3_ are crucial to support, or not, the E-EKC hypothesis. An N-shaped relationship between energy and GDP per capita is supported when ${\widehat{\beta }}_{3}>0$ and ${\widehat{\beta }}_{2}^{2}-3{\widehat{\beta }}_{3}{\widehat{\beta }}_{1}>0$. Meanwhile, there is an inverted N-shape when ${\widehat{\beta }}_{3}<0$ and ${\widehat{\beta }}_{2}^{2}-3{\widehat{\beta }}_{3}{\widehat{\beta }}_{1}>0$. The two turning points can be calculated as the exponential of the real solutions of equation ${\widehat{\beta }}_{1}+2{\widehat{\beta }}_{2}{y}_{t}+3{\widehat{\beta }}_{3}{y}_{t}^{2}=0$, adding to them the centered value of the logarithm of real GDP per capita ($\overline{Y }$). Additionally, if ${\widehat{\beta }}_{3}$ is zero and the coefficients, with respect to real GDP per capita and real GDP per capita squared, are different from zero, a U-shaped or inverted U-shaped relationship may be obtained.

#### Econometric approach to estimate Eq. (3)

On the one hand, and as mentioned previously, testing the E-EKC hypothesis requires including the explanatory variables *y*, *y*^2^, and *y*^3^, in Eq. (3). On the other hand, most previous studies, which referred to developed countries and developing countries, highlighted that GDP per capita is stationary in first differences but not in levels, therefore being integrated of order 1, that is I(1). However, as stated by Müller-Fürstenberger & Wagner (2007), and Wagner (2008, 2012, 2015), the squared and cubic values of GDP per capita are not integrated in any order. Therefore, the estimates obtained in most previous studies, which have usually used linear cointegration regression to estimate Eq. (3), may be spurious. In that sense, Wagner (2015) showed that the linear cointegration regressions approach is not adequate to estimate non-linear models, because stationary errors are serially correlated, and regressors are strongly endogenous. For these reasons, the EKC hypothesis has been supported in the most published studies (Wagner 2015; Grabarczyk et al. 2018). To address these issues, Wagner (2015) and Wagner & Hong (2016) have developed the cointegrating polynomial regressions (CPR) approach. This approach, compared to linear cointegration regressions, significantly reduces the evidence for the EKC hypothesis. For instance, Wagner (2015) tested the EKC hypothesis for 19 early industrialized countries, over the period 1870–2000, using the linear cointegration regressions and the CPR approaches. The results confirm the presence of the EKC hypothesis in 13 countries, by using the first approach, while only in four countries, using the second. Therefore, the CPR approach has been used in this study to estimate Eq. (3), as the real GDP per capita variable is I(1), and the error term (*ε*_*t*_) is found to be stationary.

**Fig. 2:**
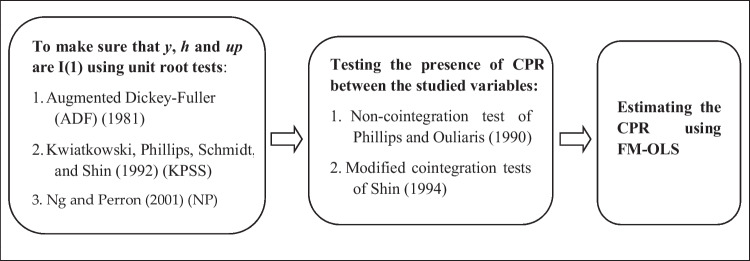
Steps of the CPR approach. ﻿ Source: Elaborated by the authors

CPR is performed sequentially, as shown in Fig. 2. In the first step, the integration order of the explanatory variables is determined not only by using the classical unit root tests, namely the Augmented Dickey-Fuller (ADF) test (Dickey & Fuller 1981), and the Kwiatkowski, Phillips, Schmidt, and Shin (KPSS) test (Kwiatkowski et al. 1992), but also by performing the efficient and the robust unit root test of Ng & Perron (2001). The time series are stationary if the non-stationary null hypothesis of the ADF and Ng and Perron tests are rejected, and the stationary null hypothesis of the KPSS test is accepted. Moreover, as stated in Grabarczyk et al. (2018), it is not necessary to test if the dependent variable, the logarithm of energy use, is stationary or not, as this is a function of a non-linear processes (a polynomial function). Meanwhile, the Lee-Strazicich unit root test (Lee & Strazicich 2003) is conducted to endogenously identify the structural changes in the time series.

In addition, the second step is to test the possible presence of CPR, between the energy use and the regressors ($y, {y}^{2}, {y}^{3},h ,up$), using the extended non-cointegration test of Phillips & Ouliaris (1990) and the modified cointegration tests of Shin (1994). There is evidence of CPR between variables, if the null hypothesis associated to the first test is rejected, and that related to the second, is accepted.

Finally, once the presence of CPR is confirmed, Eq. (3) is estimated using the extended FM-OLS to CPR, as in Wagner & Hong (2016), because the OLS estimator is not consistent in that case. Nevertheless, following Wagner & Hong (2016), and Grabarczyk et al. (2018), the OLS method is additionally performed to estimate the linear cointegration relationship, in order to compare it to CPR results. The CPR approach is presented in Fig. 2.

#### Indirect effects of human capital and energy use

The human capital indirect effects on energy use are studied through a system of simultaneous equations, as in Salim et al. (2017) and Yao et al. (2019). Additionally, a last equation is included in the system of equations, in which human capital depends on physical capital, in order to analyze the relationships of complementarity or substitutability between the factors. This system of simultaneous equations is formulated as follows:

4 $$EU=F\;\left(Y,H,UP\right)$$

5 $$Y=F\;\left(H,K,OP\right)$$

6 $$K=F\;\left(H,K\left(-1\right)\right)$$

7 $$H=F\;\left(K,H\left(-1\right)\right)$$

where *EU*, *Y*, *H*, and *UP* are already defined above; *K* is the physical capital stock per capita at constant 2011 national prices (in millions of 2011 US$) expressed in logarithms; and *OP* is crude oil prices at 2018 US$ expressed in logarithms. *K*(− 1) and *H*(− 1) are the stock of physical capital and human capital of the previous year expressed in logarithms, respectively. The data used are annual figures covering the period 1970–2017 and taken from three databases, namely World Development Indicators (World Bank 2021), Penn World Table 9.1 (Feenstra et al. 2021), and BP Statistical Review of World Energy (2020).

The production function depends on physical and human capital, according to the endogenous economic growth theories (Romer 1986, and Lucas 1988, among others), which argue that human capital also plays a large role in economic growth. Indeed, there are two main channels through which human capital may impel economic growth. Firstly, human capital, accumulated mainly from education and experience, directly affects economic growth (Lucas 1988). Secondly, human capital may indirectly impel economic growth by driving innovations and absorption of technologies, and accelerating the rate of technological change through investment in physical capital (Romer 1986, 1990; Pablo-Romero et al. 2016). In that context, the empirical study of Pablo-Romero & Sánchez-Braza (2015) not only argued that economic growth is positively linked to human and physical capital, but also that human capital reinforces its impact on growth by its complementarity with the stock of capital factor. The human and physical capital direct effects on economic growth are studied in this analysis by Eq. (5), while the complementary relationships, between both factors, by Eqs. (6) and (7).

The production function expressed in Eq. (5) also depends, in this case, on oil prices. Previous studies have been highlighting the role of oil prices in economic growth, such as in the recent study by Van Eyden et al. (2019) who find that price variability and uncertainty may be severe for oil producing countries such as Algeria. It should be noted that Algeria is the main natural gas producer in Africa, and the second supplier to Europe (U.S. Energy Information Administration 2019), being one of the countries with the highest energy production from fossil fuels in the world (Belaid & Youssef 2017). The Algerian hydrocarbon sector contributes between 30 and 50% to GDP, 97% to total exports, and 70% to tax revenue (Ainas et al. 2012; Donsimoni 2017). Thus, given the importance of the hydrocarbon sector in this country, oil prices are added as a control variable in the production function.

In order to analyze the above-mentioned relationships, Eqs. (4) to (7) are specifically expressed as follows:

8 $$E{U}_{t}={C}_{1}+{\gamma }_{1}{Y}_{t}+{\gamma }_{2}{H}_{t}+{\gamma }_{3}{UP}_{t}+{\varepsilon }_{1\mathrm{t}}$$

9 $${Y}_{t}={C}_{2}+{\gamma }_{4}{H}_{t}+{\gamma }_{5}{K}_{t}+{\gamma }_{6}{OP}_{t}+{\varepsilon }_{2\mathrm{t}}$$

10 $${K}_{t}={C}_{3}+{\gamma }_{7}{H}_{t}+{\gamma }_{8}{K}_{t-1}+{\varepsilon }_{3\mathrm{t}}$$

11 $${H}_{t}={C}_{4}+{\gamma }_{9}{K}_{t}+{\gamma }_{10}{H}_{t-1}+{\varepsilon }_{4\mathrm{t}}$$

where *γ*_1_, *γ*_2_, *γ*_3_, *γ*_4_, *γ*_5_, *γ*_6_, *γ*_7_, *γ*_8_, *γ*_9_, and *γ*_10_ are the parameters to be estimated, *ε*_1t_, *ε*_2t_, *ε*_3t_, and *ε*_4t_ are white-noise error terms.

According to Wooldridge (2010), there are two types of methods to estimate the simultaneous equations model, namely limited information methods and full information methods. The first is performed by using two-stage least squares (2SLS) and the second by using three-stage least squares (3SLS).

## Data and descriptive analysis

Data are selected from three databases, World Development Indicators of the World Bank, Penn World Table 9.1, and the BP Statistical Review. Details of the selected variables and data sources are reported in Table 2. Likewise, descriptive statistics of these data are summarized in Table 3.

**Table 2:** Studied variables and data sources

Variables	Symbol	Measure	Data source
Energy Use	EU	Kg of oil equivalent per capita	WDI^a^
Real GDP per capita	Y	Real GDP per capita at constant 2011 national prices (in mil. 2011 US$)	WPT 9.1^b^
Human Capital	H	Years of schooling and returns to education	WPT 9.1^b^
Physical Capital	K	Capital stock per capita at constant 2011 national prices	WPT9.1^b^
Urban Population	UP	Urban population ratio (as % of total population)	WDI^a^
Crudes oil prices	OP	Crude oil prices at 2018 US$	BP Statistical Review^c^

^a^World Development Indicators (World Bank 2021).

^b^Penn World Table 9.1 (Feenstra et al. 2021).

^c^BP Statistical Review of World Energy (2020)

**Table 3:** Descriptive statistics

Variable	Mean	Max	Min	Std. Dev	Jarque–Bera	Obs
*EU*	6.626	7.191	5.450	0.471	11.31	48
*Y*	9.286	9.554	8.861	0.161	0.60	48
*H*	0.480	0.832	0.157	0.220	4.34	48
*K*	10.541	10.811	10.054	0.180	17.28	48
*UP*	− 0.620	− 0.327	− 0.928	0.198	3.70	48
*OP*	3.883	4.821	2.453	0.604	1.39	48

Established by the authors

Energy use (final energy consumption) has experienced a high growth rate throughout the past five decades in Algeria, with the annual growth, over the period 1970 to 2017, being estimated at 10.67% (APRUE 2017). This strong upward trend is mainly related to changes in the way of life and the expansion of economic activity (Bélaïd and Abderrahmani 2013; Bouznit & Pablo-Romero 2016; Bouznit et al. 2018). According to the data provided by APRUE (2017), 43% of energy use is intended to meet the needs of the residential sector, while 33%, 22%, and 1% are devoted to transport, industry and agriculture, respectively.

During the period 1970–2017, the energy use trend can be divided into three periods. Over 1970 to 1980, the total energy consumption experienced a strong upward trend, with an annual growth rate of 15.02%. This period also coincided with notable economic expansion, with the annual growth rates in the first and second five-year periods of the 1970s being 7.52% and 10.37%, respectively. In the second period (1980–2000), the energy consumption grew by 41.51% over the whole period, its annual growth rate being 2.45%. Finally, the energy consumption growth rose again in the final period (2000–2017), with an annual growth rate of 4.18%. Before COVID-19, this positive trend was also expected to continue. Thus, the electricity demand was expected to more than double by 2030 (Nachmany et al. 2015).

Regarding human capital, in this study, it is measured by the indicator based on years of schooling and returns to education provided by Penn World Table 9.1 (Feenstra et al. 2021). Its value has doubled between 1970 and 2017, moving from 1.171 to 2.303, which represent an annual growth rate of 2.06%. Moreover, at the beginning of the studied period, the school enrolment rates in secondary and higher education were only 14% and 2.7%, respectively. However, in 2018, these rates had become 100% and 51.36%, respectively. Nevertheless, and although Algeria has improved its population education remarkably during the analyzed period, the educational attainment gap compared with the USA, considered as a technological frontier, has not closed.

## Results and discussion

### Estimating Eq. (3)

As mentioned previously, the CPR approach developed by Wagner (2015) and Wagner and Hong (2016) requires the study of the integration order of explanatory variables. As Table 4 and Table 5 show, the explanatory variables are I(1), according to the ADF, KPSS, and Ng and Perron unit root tests results. Therefore, CPR is appropriate to test for the existence of the non-linear relationship between the variables included in Eq. (3).

**Table 4:** Results of classical unit root tests

	In levels		In first differences		Decision
	ADF	KPSS	ADF	KPSS	
*y*	− 2.50	0.34	− 3.93***	0.13**	I(1)
*h*	− 2.65	2.46	− 3.13*	0.10***	I(1)
*up*	− 2.38	152.24	− 2.58*	0.261***	I(1)

***, **, and * indicate significance at the 1%, 5%, and 10% level, respectively. I(1): time series is stationary, after the first differences

**Table 5:** Results of the Ng and Perron unit root test

	Variables	Constant				Trend and constant				Decision
		MZa	MZt	MSB	MPT	MZa	MZt	MSB	MPT	
In levels	*y*	0.87	0.59	0.67	34.42	− 5.27	− 1.62	0.30	17.25	NS
	*h*	1.81	4.70	2.58	501.12	− 4.56	− 1.50	0.33	19.94	NS
	*up*	1.64	5.70	3.46	867.26	− 8.16	− 1.90	0.23	11.48	NS
In first diff	*y*	− 10.73**	− 2.29**	0.21*	2.36**	− 22.31**	− 3.32**	0.14**	4.20**	I(1)
	*h*	− 10.22**	− 2.23**	0.21**	2.50**	− 11.59	− 2.40*	0.20	7.86	I(1)
	*up*	− 7.30*	− 1.91*	0.26*	3.35*	− 4.22	− 1.38	0.32	20.90	I(1)

***, **, and * indicate significance at the 1%, 5% and 10% level, respectively. *NS* non stationary. I(1): time series is stationary, after the first differences

Additionally, Table 6 shows the results of the Lee-Strazicich two break points test. The latter allows us to identify several break years throughout the period, from 1980 to 2010.

**Table 6:** Results of structural changes test

	*t*-stat	Break years
*y*	-6,26	2000/2005
*h*	-5.44	1980/1996
*up*	-14.96	2003/2010

In the second step, the non-cointegration test of Phillips & Ouliaris (1990), and the cointegration tests of Shin (1994), have been performed to test if the Eq. (3) is a CPR, or not. The obtained results, shown in Table 7, indicate that there is evidence of CPR between Eq. (3) variables.

**Table 7:** Results of the non-cointegration and cointegration tests

Non-cointegration test of Phillips & Ouliaris (1990)		Cointegration test of Shin (1994)
*Z*_*t*_	*Z*_*α*_	CT
− 6.40**	− 45.24***	0.13***

*** and ** indicate significance at the 1% and 5% level, respectively

Finally, the CPR relationship is estimated by using the FM-OLS estimator, where the long-run variances are calculated by using a non-prewhitened Bartlett Kernel estimator, with a fixed Newey-West bandwidth. Additionally, in order to compare the results with those of the linear cointegration relationship (LCR), the OLS estimator was also performed. The estimated LCR was obtained by minimizing the Akaike information criterion (AIC) and Schwarz information criterion (SIC). Moreover, the errors are not serially correlated, according to the Breusch Godfrey test (LM-test) results, the null hypothesis of the presence of serial correlation [*p* value of chi-squares (2) = 0.12] being rejected. Both estimate results are reported in Table 8.

**Table 8:** Estimate results: LCR and CPR

	Linear cointegration relationship (LCR) using OLS	Cointegration polynomial regressions (CPR) using FM-OLS
*y*	0.60*	0.42**
	(1.84)	(2.31)
*y*^2^	− 2.86***	− 1.12**
	(− 4.68)	(− 2.14)
*y*^3^	− 3.83*	4.33**
	(− 1.67)	(2.38)
*h*	− 2.55***	− 2.65***
	(− 2.56)	(− 3.44)
*up*	1.28	4.13***
	(1.07)	(4.24)
*c*	− 1.21***	− 0.14***
	(− 2.64)	(− 2.55)
*D*1980	0.21***	0.25***
	(3.17)	(3.99)
*D*2000	− 0.23***	**–**
	(− 3.96)	**–**
*t*	0.05***	**–**
	(2.70)	**–**
E-EKC	**Yes** inverted N-shape curve	No
Turing points	**6029/7547** US$	**–**
Nb. Obs	48	47
AIC	2.37	**–**
SC	− 2.02	**–**
Long-run variance	**–**	0.004
*R*^2^	0.98	0.97

*t* value in brackets; ***, **, and * indicate significance at the 1%, 5%, and 10% level, respectively

The first column in Table 8 shows the LCR results using the ordinary least squares method (OLS). The estimated coefficient related to the real GDP per capita is positive, and statistically significant, at 10%. Meanwhile, the estimated coefficients, associated to the squared and cubic real GDP, present negative and significant values. As $${\widehat{\beta }}_{3}<$$ 0, and $${\widehat{\beta }}_{2}^{2}-3{\widehat{\beta }}_{3}{\widehat{\beta }}_{1}>$$ 0 [i.e., − 3.83 < 0 and (− 2.86)2 − 3*(− 3.83)*0.60 = 8.17 + 6.89 = 15.06 > 0], the relationship between energy use and real GDP per capita is described as an inverted N-shape curve, the turning points being equal to US$ 6029/US$ 7547. Nevertheless, as mentioned above, this result may be spurious, as GDP per capita is I(1) and introducing its squared and cubed terms in the LCR may generate endogeneity (Wagner, 2015). Therefore, the robustness of this result should be checked using the cointegration polynomial regressions (CPR) approach.

**Fig. 3:**
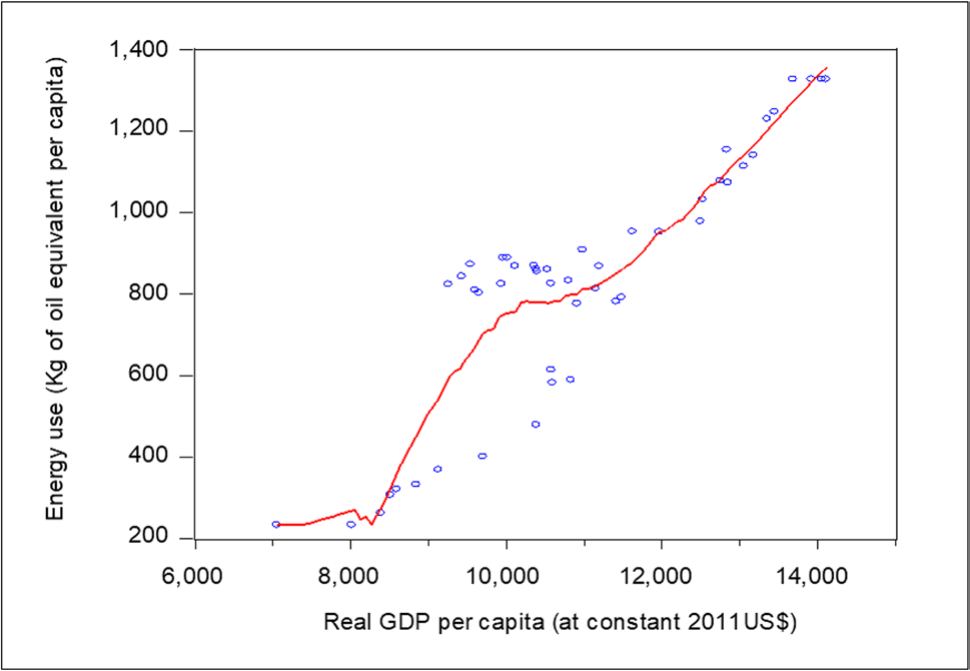
Scatter plot: Relationship between income and energy demand in Algeria. Red line represents the polynomial fit

To solve this problem, the Eq. (3) has been re-estimated, according to the CPR approach, by using FM-OLS. As shown in the third column in Table 8, the coefficient related to real GDP per capita is positive and statistically significant, implying a positive effect on the energy use per capita. The latter will increase by 0.42%, if the real GDP per capita enhances by 1%. This positive relationship, between real GDP per capita and energy consumption, is consistent with the results obtained in Narayan & Smyth (2008), Apergis & Payne (2010), Lee & Chien (2010), Azam et al. (2015), Salim et al. (2017), Bouznit et al. (2018), and Yao et al. (2019). Furthermore, the estimated coefficients related to real GDP per capita squared, and real GDP per capita cubed, are statistically significant, and their signs are negative and positive, respectively. Therefore, there is either an N-shaped relationship, or a monotonic increasing relationship, between total energy consumption and economic growth. As ${\widehat{\beta }}_{3}$ > 0, and ${\widehat{\beta }}_{2}^{2}-3{\widehat{\beta }}_{3}{\widehat{\beta }}_{1}$ < 0 [i.e., 4.33 > 0 and (− 1.12)^2^ − 3*4.33*0.42 = 1.25 – 5.45 =  − 4.2 < 0], and according to Fig. 1, the relationship between income and energy demand in Algeria is a monotonic increasing relationship. Therefore, the LCR results which indicate the presence of inverted N-shape are not robust, and the Energy-EKC hypothesis is not supported, as the CPR approach results indicate. Therefore, more energy use, in per capita terms, can be expected as GDP grows, leading, in turn, to accentuate the CO_2_ emission in the coming years. This result is in concordance with the scatter plot between real GDP per capita and energy demand in Algeria, as shown in Fig. 3. It is therefore convenient to apply more energy policies tending to control the growth in energy consumption, and/or alternatively introduce more renewable energies, to avoid this growth translating into more emissions. Indeed, the absence of the Energy-EKC hypothesis means that Algeria has not yet reached the development threshold which could lead to negatively affect carbon emissions. In fact, electricity consumption is expected to move from 80 TWh in 2020 to 150 TWh in 2030, representing a growth rate of 87.5% (Bouznit et al. 2022). Therefore, with the share of fossil fuel in the total electricity generation estimated at more than 98% in 2022, the CO_2_ emission in Algeria will continue to increase over time. Thus, policymakers in Algeria should promote suitable factors that can reduce the dirty energy consumption with a view to enhancing the environmental framework.

The results also show that human capital negatively impacts on total energy consumption. Specifically, a 1% increase in years of schooling and returns to education will decrease energy consumption by 2.65%. This result is in line with those obtained by Salim et al. (2017), Shahbaz et al. (2019), and Yao et al. (2019). Thus, promoting human capital is a good way to control the growth in energy use. Indeed, a high level of human capital leads to an increase in the innovation and technological progress necessary to promote the generation of renewable energy and enhance energy saving. Further, well-educated people tend to use clean energy, leading to the consumption of less energy (Jacksohn et al. 2019; Yao et al. 2019).

In addition, the estimate findings also reveal that urbanization plays a positive role in total energy consumption. An increase of 1% in urban population leads to a rise in total energy consumption of 4.13%. Indeed, the expansion of urbanization significantly increases housing electricity demand, transport network and traffic, and industry activity, which lead to increase energy consumption. In that sense, Wang et al. (2020b) suggested that urbanization, in the developing countries in Asia, the Middle East, and North Africa, is one of the determinants of residential energy consumption.

### Indirect effect of human capital

The system of simultaneous Eqs. (8) to (11) is estimated by using 2SLS and 3SLS. The obtained results are reported in Table 9. Columns A to D report the results obtained by using 3SLS, while columns E to H are those obtained by using 2SLS. The results do not differ substantially between both methods.

**Table 9:** Estimating the system of simultaneous equations using 3SLS and 2SLS methods

	3SLS				2SLS			
	A	B	C	D	E	F	G	H
	*EU*	*Y*	*K*	*H*	*EU*	*Y*	*K*	*H*
*C*	3.18	4.81***	0.36*	− 0.25***	8.34***	5.89***	0.59***	− 0.31***
	(3.02)	(0.65)	(0.21)	(0.06)	(3.39)	(0.71)	(0.22)	(0.07)
*Y*	1.03***				0.62**			
	(0.224)				(0.27)			
*H*	− 4.57***	0.29***	− 0.02		− 5.56***	0.32***	− 0.01	
	(0.87)	(0.04)	(0.01)		(0.96)	(0.05)	(0.01)	
*UP*	6.45***				7.81***			
	(1.06)				(1.17)			
*OP*		0.08***				0.12***		
		(0.01)				(0.01)		
*K*		0.37***		0.02***		0.25***		0.03***
		(0.06)		(0.006)		(0.07)		(0.007)
*H*(− 1)				0.99***				0.98***
				(0.005)				(0.005)
*K*(− 1)			0.96***				0.94***	
			(0.02)				(0.2)	
R^2^	0.91	0.86	0.98	0.99	0.92	0.87	0.98	0.99
Obs	47	47	47	47	47	47	47	47

Standard errors in brackets; ***, ** and * indicate significance at the 1%, 5%, and 10% level, respectively

Columns (A) and (E) in Table 9 show that the elasticity, with respect to real GDP per capita, is positive and statically significant. This result is in line with those obtained by Azam et al. (2015), Salim et al. (2017), Bouznit et al. (2018), and Yao et al. (2019). Likewise, the results related to urban population, contained in column (A) and column (E), show a positive effect on energy consumption. Nevertheless, the estimated elasticity value is higher than that related to real GDP per capita. Finally, the results related to human capital are similar to those obtained in the previous long-run estimate. A significant negative impact is observed.

Equation (9) estimation results are shown in columns (B) and (F) in Table 8. Estimated human capital elasticity is positive and statistically significant, implying a positive relationship between it and real GDP per capita, as stated by the endogenous models. Additionally, this positive effect of human capital on economic growth in Algeria is also observed in the studies by Mekdad et al. (2014), Abderrahmane et al. (2016), and Bentoumi and Gaidi (2020). However, contrary to other studies which do not find causality between variables, as in Boutayeba and Ramli (2019), who consider that this lack of causality could be related to the lack of educational level, more than lack of human capital quantity.

The positive impact of human capital on GDP, observed in the results, also implies that human capital has an indirect positive effect on energy use. According to columns (B), a 1% increase in human capital raises real GDP per capita by 0.29%, thereby the energy consumption tends to grow by 0.30% (0.29*1.03%). This increase is lower than that of the direct estimated effect.

Equation (9) estimation results also show that a 1% increase in physical capital stock, per capita, leads to enhancing real GDP per capita by 0.37%, and in turn the energy consumption by 0.38%. It is also worth noting that oil prices impact positively on real GDP.

**Fig. 4:**
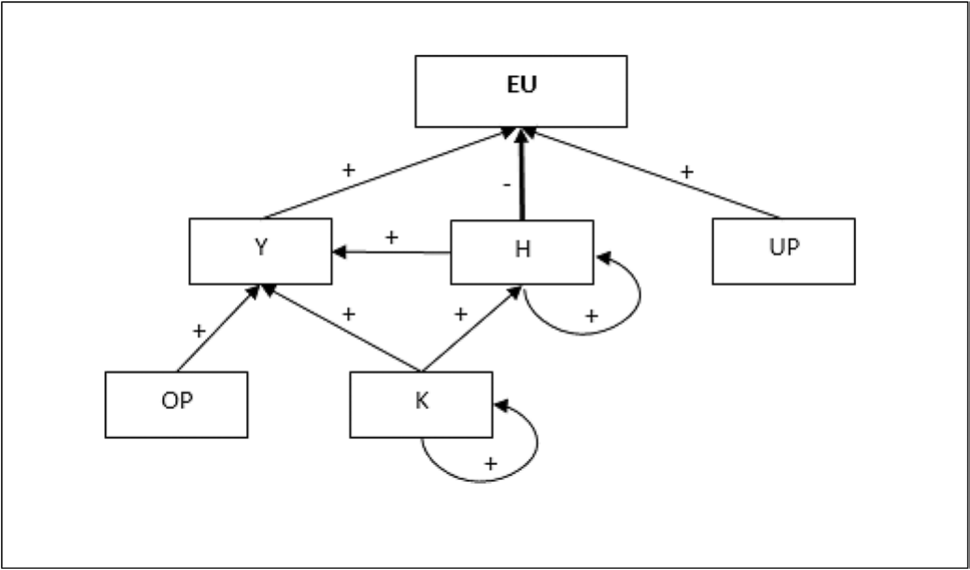
Direct and indirect effects of human capital and other variables on energy use

The estimated results of Eq. (10), which are recorded in column (C) and column (G), show that the physical capital is only significantly influenced by the previous stock of capital. However, the estimated Eq. (11), column (D) and (H), confirm that stock of physical capital per capita positively affects human capital, implying a complementarity relationship between them. This result confirms the hypothesis in Griliches (1969) and Pablo-Romero and Sánchez-Braza (2015). The results show that the physical capital positively influences energy consumption, via the human capital channel, because human capital promotes economic growth and this growth induces the energy demand increase. Nevertheless, the amplitude of this effect is very weak: a 1% increase in physical capital induces human capital by 0.02%, and, therefore, the energy consumption by 0.006%. However, it is also worth noting that physical capital negatively influences energy consumption via the human capital, because human capital has a significant negative effect on energy consumption. In this case, the effect is greater, as a 1% increase in physical capital increases human capital by 0.02%, and, therefore, an energy consumption reduction of 0.09% (0.02 × 4.57). Thus, the increase in capital will induce a reduction in energy consumption via human capital increase. In spite of this, the increase in physical capital has a positive effect on economic growth, which in turn increases energy consumption. The results show that this energy increase effect via economic growth is greater than the energy decrease via human capital. Thus, the results show that physical capital increases generate energy consumption increases. All these relationships between variables are summarized in Fig. 4.

The joint results of the direct and indirect effects of human capital on energy consumption reveal that, human capital increases reduce the energy consumption in Algeria, it is therefore appropriate to promote reduction. This promotion could have two positive effects. On the one hand, economic growth could be enhanced, while on the second, energy consumption could be slowed, helping to control emissions. However, as previous research by Boutayeba & Ramli (2019), and others, has pointed out the need to improve the quality of education, rather than increasing its quantity, these positive effects on the reduction of energy consumption may also depend on this improvement in the quality of human capital. In this sense, the low values of the Algerian education system in the PISA-2015 ranking, by OECD (2018), should be highlighted. Thus, for example, the indicator of the average achievement in science reached the value of 376 in Algeria, while the OECD average reached the figure of 493. This score has placed it in the penultimate position of the report’s list. In that sense, the long-term potential for quality educational improvements is great and, therefore, the possibilities for growth and energy reduction.

## Conclusions and policy implications

This article analyzes the direct and indirect effects of human capital on energy consumption in Algeria, over the period 1970–2017. The direct effect of human capital was studied by analyzing the E-EKC hypothesis, extended with human capital and urban population. The methodology adopted is the CPR approach with break points. The indirect effect is studied by using a simultaneous equations model that defines the productive relationships, between production, human capital, and physical capital. The empirical results confirm the presence of direct and indirect effects of human capital on energy consumption.

Regarding the direct effects, the CPR results reveal that human capital negatively impacts total energy consumption more that proportionally, indicating that focusing on promoting human capital is appropriate to control energy use. These measures are specially recommended in a context of income growth, as the E-EKC hypothesis is not supported in Algeria. In fact, a monotonic increasing relationship is observed between the energy and income variables. Likewise, the estimated results also show a positive effect of urbanization on energy consumption, which could be related to the residential energy consumption increase, observed in Algeria. Consequently, increasing energy demand could be expected in the economic growth process. The energy growth associated with Algeria’s economic growth makes it desirable to take measures aimed at guaranteeing such consumption in the future. Also, given the high consumption of fossil fuels in Algeria, it may be desirable to promote the increased use of renewable energy sources if emissions growth is to be contained.

The result of the system of simultaneous equations estimates indicates that human capital positively affects GDP, implying a positive indirect effect on energy use. However, this increase is lower than that of the direct estimated effect; therefore, the total effect of human capital on energy consumption is negative. Consequently, a human capital promotion could have two positive effects, by enhancing economic growth and controlling energy consumption. However, noting the PISA results (OECD 2018) and results of previous literature, it could be more appropriate to promote the increase in human capital quality, instead to human capital quantity. This could have a greater impact on economic growth and even on the control of the growth of energy demand.

On the other hand, the results also show that physical capital increases GDP, and therefore energy consumption, indirectly. However, two other indirect effects of physical capital on energy demand are observed. Firstly, physical capital positively affects human capital and indirectly and positively affects economic growth, and therefore energy consumption. However, this effect is very weak. Secondly, the increase in physical capital induces a reduction in energy consumption, via the direct effect of human capital increase. Considering all these effects of physical capital on energy consumption, the result is a positive impact of physical capital on energy consumption. In that sense, an economic growth focused more on the human capital factor, than on the physical capital, could reduce the effects of economic growth on increases in energy demand.

Two main policy measures can be advised after this work. On the one hand, energy growth associated with economic growth makes it appropriate to adapt the energy system to growing demand, promoting greater use of renewable energies, for example solar, if emissions growth is to be contained. On the other hand, improving the human capital can help contain energy growth, in turn promoting more sustainable consumption.

## Data Availability

Not applicable.
